# Maturation of molybdoenzymes and its influence on the pathogenesis of non-typeable *Haemophilus influenzae*

**DOI:** 10.3389/fmicb.2015.01219

**Published:** 2015-11-05

**Authors:** Rabeb Dhouib, Dk S. M. Pg Othman, Ama-Tawiah Essilfie, Phil M. Hansbro, Jeffrey O. Hanson, Alastair G. McEwan, Ulrike Kappler

**Affiliations:** ^1^Centre for Metals in Biology, Australian Infectious Diseases Research Centre, School of Chemistry and Molecular Biosciences, The University of QueenslandSt. Lucia, QLD, Australia; ^2^Centre for Asthma and Respiratory Diseases and Hunter Medical Research Institute, The University of NewcastleNewcastle, NSW, Australia; ^3^School of Biological Sciences, The University of QueenslandSt. Lucia, QLD, Australia

**Keywords:** Haemophilus influenzae, molybdenum cofactor, enzyme maturation, biofilm formation, cell interactions

## Abstract

Mononuclear molybdenum enzymes of the dimethylsulfoxide (DMSO) reductase family occur exclusively in prokaryotes, and a loss of some these enzymes has been linked to a loss of bacterial virulence in several cases. The MobA protein catalyzes the final step in the synthesis of the molybdenum guanine dinucleotide (MGD) cofactor that is exclusive to enzymes of the DMSO reductase family. MobA has been proposed as a potential target for control of virulence since its inhibition would affect the activities of all molybdoenzymes dependent upon MGD. Here, we have studied the phenotype of a *mobA* mutant of the host-adapted human pathogen *Haemophilus influenzae*. *H. influenzae* causes and contributes to a variety of acute and chronic diseases of the respiratory tract, and several enzymes of the DMSO reductase family are conserved and highly expressed in this bacterium. The *mobA* mutation caused a significant decrease in the activities of all Mo-enzymes present, and also resulted in a small defect in anaerobic growth. However, we did not detect a defect in *in vitro* biofilm formation nor in invasion and adherence to human epithelial cells in tissue culture compared to the wild-type. In a murine *in vivo* model, the *mobA* mutant showed only a mild attenuation compared to the wild-type. In summary, our data show that MobA is essential for the activities of molybdenum enzymes, but does not appear to affect the fitness of *H. influenzae*. These results suggest that MobA is unlikely to be a useful target for antimicrobials, at least for the purpose of treating *H. influenzae* infections.

## Introduction

Mononuclear molybdenum enzymes occur in all known forms of life (Zhang and Gladyshev, 2008) where they catalyze important oxidation-reduction reactions, particularly those associated with bacterial respiration and energy conversion processes. Several recent reports have linked molybdenum enzymes to virulence in a variety of bacteria. In *Mycobacterium tuberculosis*, it has been observed that mutants lacking respiratory nitrate reductase (Nar) exhibit reduced persistence in the organs of immuno-deficient mice (Aly et al., 2006). In the gut, *Salmonella enterica* sv Typhimurium uses tetrathionate, generated from sulfide as a consequence of the macrophage respiratory burst, in anaerobic respiration using the molybdoenzyme tetrathionate reductase (Ttr) and the presence of this enzyme appears to confer a selective advantage during colonization of the inflamed gut mucosa (Winter et al., 2010).

Nar and Ttr are members of the DMSO reductase family of molybdenum enzymes, a large and expanding enzyme class that is uniquely found in prokaryotes (Magalon et al., 2011). A distinguishing trait of these enzymes is that they contain a modified version of the molybdopterin (MPT) organic component of the molybdenum cofactor that is common to all mononuclear molybdoenzymes (Leimkühler et al., 2011). While all known mammalian molybdoenzymes contain Mo coordinated by a single MPT, in the bacterial DMSO reductase family enzymes, the Mo ion is coordinated by two molecules of MPT modified by the addition of a nucleotide and known as molybdopterin guanine dinucleotide (MGD) (Schwarz et al., 2009). The addition of the guanine to the basic Mo-MPT unit is catalyzed by the MGD biosynthesis protein MobA (molybdenum cofactor guanylyltransferase) and may also be influenced by the MobB protein (Leimkühler et al., 2011). Studies analyzing the function of MobB or effects of *mobB* gene mutations, however, showed that MobB does not appear to play an essential role in the synthesis of MGD (Eaves et al., 1997; Buchanan et al., 2001).

In contrast, mutations of the *mobA* gene have been shown to have pleiotropic effects on the activities of molybdoenzymes in *E. coli* (Johnson et al., 1991; Palmer et al., 1994), *Rhodobacter capsulatus* (Leimkuhler and Klipp, 1999), *Rhodobacter sphaeroides* (Buchanan et al., 2001), and *Pseudomonas aeruginosa* (Noriega et al., 2005) and can lead to an accumulation of the precursor Mo-MPT cofactor (Palmer et al., 1996).

The fact that the MGD containing form of the Mo cofactor is not found in human molybdenum enzymes (such as xanthine oxidase) has led to the suggestion that MobA could be a potential target for antimicrobial therapy (Anishetty et al., 2005; Williams et al., 2014), as it would affect the activities of all DMSO reductase family enzymes at the same time and thus could potentiate the already striking effects that have been observed for single gene knockouts.

*H. influenzae* possesses four respiratory molybdenum enzymes of the DMSO reductase family: formate dehydrogenase, DMSO reductase (DmsABC), a putative TMAO reductase (TorZ), and a periplasmic nitrate reductase (Nap) (Othman et al., 2014). Non-typeable *Haemophilus influenzae* (NTHI) causes and contributes to diseases such as otitis media, conjunctivitis, sinusitis, and lower respiratory tract infections in individuals with chronic obstructive pulmonary disease (COPD) and cystic fibrosis (Foxwell et al., 1998; Costerton et al., 1999; Post et al., 2000; Moghaddam et al., 2011). One molybdenum enzyme (DMSO reductase) was found to be highly expressed during interaction of NTHI with human respiratory tract-derived epithelial cells (van Ulsen et al., 2002), suggesting a link to colonization and persistence of NTHI in the respiratory tract. Also, in *Actinobacillus pleuropneumoniae*, another member of the Pasteurellaceae family of bacteria, it has been observed that mutants lacking DMSO reductase were unable to cause pneumonia in pigs (Baltes et al., 2003).

Given the central role of MobA in the biogenesis of Mo enzymes, we have assessed the effect of a *mobA* mutation on the growth physiology of NTHI, on its interactions with epithelial cells and neutrophils and on its virulence using a mouse model of infection. The underlying hypothesis for this work was that since Mo-enzymes appear to play a role in host colonization and pathogenesis in many bacteria including Pasteurellaceae, a removal of *mobA* should lead to a loss of all Mo-enzyme activities and reduced virulence.

## Materials and methods

### Bacterial strains and growth conditions

NTHI 2019 (Campagnari et al., 1987) wild-type (HI2019^WT^) and derivatives of this strain were cultivated on supplemented brain heart infusion (sBHI) agar (Becton Dickinson) that contained 10 μg/mL hemin (Johnston, 2010) and 10 μg/mL β-NAD at 37°C with 5% CO_2_. A chemically defined growth medium (sRPMI) was also used and contained 25 mM HEPES, pH 7.3, 0.8 mM sodium pyruvate, 0.08 mg/mL uracil, 0.17 mg/mL inosine, 8 μg/mL β-NAD, 17 μg/mL hemin, and 2 mg/mL NaHCO_3_ in RPMI1640 (Sigma Aldrich; Coleman et al., 2003). *E. coli* DH5α (Life Technologies) was grown in Luria Bertani (LB) broth or on LB agar (Sambrook et al., 1982) at 37°C. Kanamycin (kan) (100 μg/mL *E. coli*; 10 μg/mL NTHI), spectinomycin (spec) (50 μg/mL *E. coli*; 20 μg/mL NTHI), and ampicillin (100 μg/mL *E. coli*) were added to culture media when required.

### Growth experiments

Bacterial growth in liquid medium was determined using sRPMI and three different oxygen concentrations (Cooper et al., 2003; Othman et al., 2014). Aerobic and microaerophilic cultures were incubated at 37°C at 200 rpm using a culture volume of 25 mL and 150 mL in sterile 250 mL Erlenmeyer flasks with screw cap closures, respectively. Anaerobic cultures were incubated without agitation at 37°C in a CO_2_ (5%) enriched atmosphere in a sealed and completely filled 50 mL falcon tube. For growth experiments, bacteria grown on fresh sBHI-agar plates overnight were scraped using sterile loop, re-suspended in 5 mL sRPMI, and then used to inoculate 50 mL of sRPMI to an initial OD_600nm_ value of 0.07. After being incubated overnight at 37°C with 5% CO_2_, precultures were used to inoculate the main cultures to an initial OD_600nm_ of 0.07.

### Construction and complementation of a HI2019^Δ*mobA*^ mutant

Two fragments (about 600 bp each) covering the *mobA* gene were amplified from HI2019 genomic DNA, isolated with the kit Genomic DNA mini kit (Life Technologies), using GoTaq® Green Master Mix (Promega) and the following couple of primers: i) HI_mobA_extF and HI_mobA_intR and ii) HI_mobA_extR and HI_mobA_intF (Table 1). The primers were designed using the *Haemophilus influenzae* (HI) strain RdKW20 genome (Fleischmann et al., 1995), as the genome sequence of HI2019 was not available at the time this study started, and allow the insertion of an internal *Bam*HI restriction site in *mobA* gene. The obtained fragments were digested with *Bam*HI as well as a kanamycin (kan) resistance cassette that was amplified from the pUC-4K plasmid (Vieira and Messing, 1982) using Kan-BamHI-F and Kan-BamHI-R as primers (Table 1) before being cloned together using a three way ligation into pGEM®T-easy (Promega) to create pGEM-*mobA::Kan*.

**Table 1:** **Oligonucleotide primers used in this study**.

**Primers**	**Sequences**
HI-mobA-extF	5′- TAA TGC GCT CAC GCC AGC -3′
HI-mobA-extR	5′- GCT CAT ATT TAT CTG TACC -3′
Kan-BamHI-F	5′- AAAA GGA TCC GGA AAG CCA CGT TGT GTC -3′
Kan-BamHI-R	5′- AAAA GGA TCC CTG AGG TCT GCC TCG TGA -3′
HI-mobA-intF	5′- AAAA GGA TCC GAG TTT ATC TAA AAG ATT CA -3′
HI-mobA-intR	5′- AAAA GGA TCC AAA AGT GCG GTT AAA AAT G -3′
HI-compl-mobAF	5′- AAAA CCC GGG TTA GAT GAC TGT TTT C -3′
HI-compl-mobAR	5′- AAAA CCC GGG GAT GAC AAT TAC AAT AA -3′

The resulting plasmid was linearized with *Sac*I and transformed into competent HI2019 (Poje and Redfield, 2003) generating HI2019^Δ*mobA*^ following a selection on sBHI + 10 μg/mL kan agar plates. The inactivation of *mobA* was confirmed by PCR.

To complement the HI2019^Δ*mobA*^ mutant, the *mobA* gene (579 bp) was amplified using the primers HI_compl_mobAF and HI_compl_mobAR (Table 1) and cloned into the p601.1-Sp2 (Johnston et al., 2007) using the *Xma*I site. The resulting construct was linearized using *Bam*HI and transformed into competent HI2019^Δ*mobA*^ generating HI2019^Δ*mobA*_comp^ following a selection on sBHI + 10 μg/mL Kan + 20 μg/mL spec agar plates. Correct integration of the construct was confirmed by PCR.

### Quantitative RT-PCR

Quantitative RT-PCR (qRT-PCR) was performed as described by Othman et al. (2014) with slight modifications. Briefly, RNA was isolated from 2 mL of HI2019^Δ*mobA*^ cultures grown in triplicates to an OD_600nm_ of 0.4 for anaerobic conditions and 0.8 for aerobic and microaerophilic conditions using the GE Healthcare Illustra RNA spin Mini Kit according to the manufacturer's instructions. After removing the gDNA that contaminates the RNA samples using the Turbo DNA-free™ (Life Technologies), the biological replicates for each condition were combined and cDNA was synthesized from 500 ng of RNA using the Superscript III Reverse Transcriptase (Life Technologies). RNA concentrations were determined using the Quant-IT RNA kit (Life Technologies). qRT-PCR reactions (10 μL) used diluted cDNA (1:100−1:10000) as template, the SYBR green Mastermix (Applied Biosystems), and primers described previously (Othman et al., 2014). The 16 S rDNA gene was used as the reference gene and data analysis and normalization was performed as in (Kappler et al., 2005).

### Enzyme assays

NTHI strains were grown under anaerobic or microaerophilic conditions in 150 mL of sRPMI. Bacteria were harvested at 2300 × *g* for 10 min at 4°C and cell pellets stored at –20°C before being used. Cells were disrupted using BugBuster® Master Mix (Novagen) and insoluble components were removed by centrifugation at 13000 × *g* (15 min, room temperature). The resulting supernatant (crude extract) was collected and used for enzyme assays.

Sulfoxide and nitrate reductase activities were assayed at 37°C by monitoring the oxidation of reduced benzyl viologen at 600 nm (ε_600_ = 7.4 mM^−1^ cm^−1^; Dickie and Weiner, 1979). Enzymes assays were performed anaerobically in 20 mM sodium phosphate buffer (pH 6.8) containing 0.2 mM benzyl viologen, 0.3 mM sodium dithionite, and one of the following terminal electron acceptors: 17 mM DMSO, 5 mM DL-methionine sulfoxide (MetSO), or 10 mM potassium nitrate.

Formate dehydrogenase activity was determined anaerobically at 600 nm in assays containing 75 μM of dichlorophenolindophenol (DCPIP; ε_600_ = 21 mM^−1^ cm^−1^) in 20 mM sodium phosphate buffer (pH 6.8), 0.25 mM phenazine metasulfate, and 20 mM sodium formate as electron acceptor (Enoch and Lester, 1982). Specific activities are given as μmol of substrate reduced or oxidized per min (U) and mg of protein present. Protein concentrations were determined using the BCA-1 kit (Sigma-Aldrich).

### Tissue culture

Human bronchial epithelial 16HBE14 cells (Gruenert et al., 1988), kindly provided by Dr Kirsten Spann (Queensland University of Technology), were propagated in MEM - GlutaMAX™ (Gibco®, Life Technologies), supplemented with 10% fetal calf serum (Gibco®, Life Technologies, Cat.-No. 16000-044; sMEM). The cells were seeded into individual wells of 24-well culture dishes (Greiner Bio-One, Cat.-No. 662160) at an approximate density of 2^*^10^5^ cells/well, incubated at 37°C with 5% CO_2_ until reaching confluence, then used for infection studies.

### Adherence and invasion assays

Bacterial invasion was determined using a standard gentamycin-survival assay (St Geme and Falkow, 1990). Fresh overnight cultures of HI2019^WT^, HI2019^Δ*mobA*^, and HI2019^Δ*mobA*_comp^ were prepared on sBHI-agar plates. The bacteria were resuspended in 5 mL sMEM and then diluted in the same culture medium to 2^*^10^7^ bacteria/mL. Confluent 16HBE14 monolayers in 24-well culture dishes were washed once with fresh pre-warmed sMEM and infected using a multiplicity of infection (MOI) of 1:100 (epithelial cells: NTHI strains). The infected monolayers were incubated for 4 h or 24 h at 37°C with 5% CO_2_, washed three times with pre-warmed sMEM before sMEM containing gentamycin (50 μg/mL) was added followed by an incubation for 1 h at 37°C, 5% CO_2_. The monolayers were washed three times with fresh sMEM and lysed by the addition of sterile 1% (w/v) saponin in 1 × PBS (pH 7.4). The epithelial cell lysates were mixed thoroughly by vigorous pipetting and serially diluted in BHI broth. Dilutions (5 μL of 10^0^ to 10^−6^ dilutions) were plated on sBHI agar and incubated overnight to estimate the numbers of colony-forming units (CFU) per well. Experiments determining bacterial adherence and invasion were carried out in the same way but omitting the gentamicin incubation.

### Immunofluorescence staining

The 16HBE14 cells were grown to confluence on glass coverslips (13 mm, Number#1, ProSciTech), placed in 24-well plates (Greiner Bio-One, Cat.-No. 662160), and then infected with NTHI strains as described for adherence and invasion assays. After 4 or 24 h of incubation at 37°C with 5% CO_2_, planktonic cells were removed by washing three times with 1 × PBS. Epithelial cells and bacteria were then fixed in 4% paraformaldehyde for 15 min, permeabilized with 0.1% Triton X-100 in 1 × PBS for 5 min, and blocked overnight at 4°C with blocking buffer (2% BSA, 0.02% sodium azide in 1 × PBS). Immunofluorescence staining of NTHI was performed using the primary antibody 6E4 (200 μL of 1:100 dilution) kindly provided by Prof. Michael Jennings (Institute for Glycomics, Griffith University, Australia; Erwin et al., 2006). After 3 h of incubation at room temperature, the wells were washed three times with 500 μL of blocking buffer before addition of 200 μL of a 1:100 dilution of the secondary antibody Anti-mouse IgG (whole molecule)-FITC antibody produced in goat (Sigma Aldrich) and incubation for 2 h in the dark. Epithelial cells were stained with CellTracker™ Orange CMTMR fluorescent dye (Life Technologies; 200 μL of 1 μg/mL solution) for 1 h at room temperature in the dark, coverslips were mounted onto slides using ProLong® Gold antifade reagent (Life Technologies), and images were acquired using an Axiophot 2 epifluorescence light microscope (Zeiss).

### Neutrophil killing assays

Human neutrophils were isolated and purified from venous blood using the PolyMorphPrep kit (Axis-Shield) as per the manufacturer's instructions and seeded into 96-well plates at 2^*^10^5^ cells/well. NTHI strains grown overnight on fresh sBHI-agar plates were resuspended in RPMI medium containing 2% heat inactivated autologous human plasma, diluted to 2^*^10^7^ CFU/mL in the same medium and then added to neutrophils at an MOI of 1:10 (neutrophils: NTHI strains; Walker et al., 2007). Plates were centrifuged at 500 × *g* for 10 min then incubated at 37°C with 5% CO_2_ for 2 h. After incubation, neutrophils were lysed with water, the content of each well serially diluted in BHI and plated on sBHI-agar for overnight incubation and enumeration of CFU. *E. coli* Dh5α (Life Technologies) was used as a positive control. Percent survival of bacteria was calculated as [(CFU/ml experimental well)/(CFU/ml initial inoculum)]^*^100%.

### Murine model infection assays

Experimental animal procedures were carried out in strict accordance with the recommendations in the NSW Animal Research Regulation 2005, and the Australian code of practice for the care and use of animals for scientific purposes of the National Health. Protocols were approved by the Animal Care and Ethics Committee of the University of Newcastle and the University of Queensland.

For NTHI pulmonary infection, a mouse model described by Morey et al. (2013) was used. NTHI strains were grown in sBHI for 16 h at 37°C with 5% CO_2_. BALB/c female mice (5 to 6 weeks old) were inoculated intranasally with 30 μL of a bacterial suspension containing 10^7^ CFUs. Groups of six mice were euthanized and necropsied at 0, 24, 48, and 72 h.

To quantify the bacterial recovery, lungs were aseptically removed, homogenized in 1 mL 1 × PBS and serially diluted in the same buffer. Each dilution was plated onto sBHI plate incubated overnight at 37°C with 5% CO_2_ and CFUs per lung were calculated (Essilfie et al., 2011).

Statistical comparisons of mean CFU/lung were performed by One-way ANOVA using the Tukey *post hoc* method as integrated into the Prism 6 software package. A *p* < 0.05 was considered statistically significant.

## Results

### MobA is highly conserved within *H. influenzae* strains

**Figure 1:**
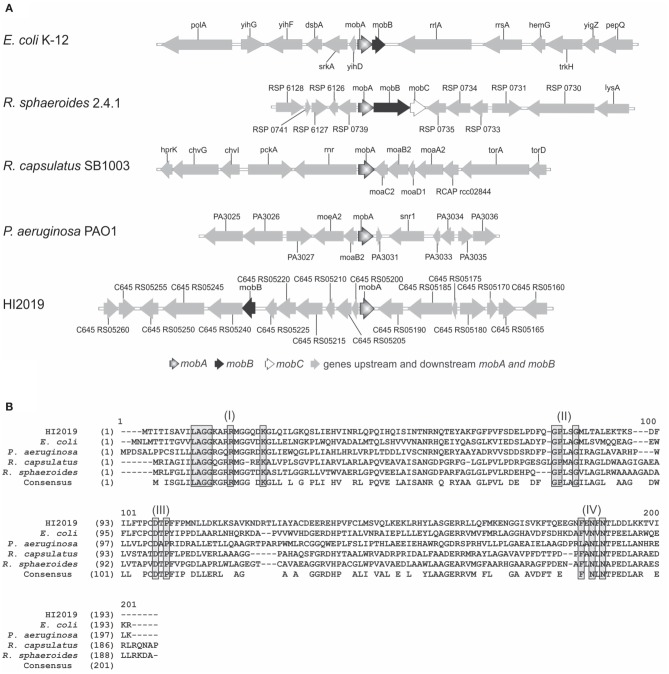
**Comparison of ***mobA*** gene loci and MobA protein sequences**. **(A)** Genomic comparison of *mob* locus in different strains: *E. coli* K-12, *Rhodobacter sphaeroides* 2.4.1, *Rhodobacter capsulatus* SB1003, *Pseudomonas aeruginosa* PAO1 and HI2019. **(B)** Alignment of MobA amino acid sequences from various bacteria. Alignments of MobA sequences from HI2019 (C645_RS05195), *E. coli* strain K-12 (Y75_p3328), *Rhodobacter sphaeroides* strain 2.4.1 (YP_353815), *Pseudomonas aeruginosa* strain PAO1 (PA3030), and *Rhodobacter capsulatus* strain SB1003 (RCAP_rcc02839) using the ALignX module of Vector NTI® software 11.5.2 (Life Technologies). Motifs predicted to be required for substrate interactions (Lake et al., 2000) are boxed in gray and labeled I to IV.

We previously reported the presence of several molybdenum enzymes in the *H. influenzae* respiratory chain (Othman et al., 2014). In keeping with this and earlier work (Stevenson et al., 2000), *mobA* and *mobB* genes were present within all *H. influenzae* strains (Table S1). Analysis of the genomes of HI2019, HI RdKW20, seven other NTHI strains (NTHI 86-028NP, NTHI R2846, NTHI F3047, NTHI F3031, NTHI PittEE, NTHI R2866, and NTHI PittGG) and two encapsulated *H. influenzae* strains (HI 10810 and HI KR494, serotypes b and f) revealed that the two genes do not form a transcriptional unit and are separated by six conserved ORFs in all strains except NTHI R2866 and HI KR494 strains where seven intervening genes are present. The six ORFs separating *mobA* and *mobB* encode a protein related to the RseC extracytoplasmic sigma factor regulatory protein, a SAM-dependent methyltransferase (YhiQ), a tRNA methyltransferase (TrmA), a hypothetical protein (YifE/DUF413 group), a Thiol:sulfide interchange protein (DsbA), and another hypothetical protein (YihD family/DUF1040 group; Figure S1). This gene arrangement is unique and differs from that found in other bacteria where *mobA* function has been studied (Figure 1A).

Within *H. influenzae* strains, MobA and MobB amino acid identity values are between 97 and 100%, except for MobA from strains HI KR494 and HI 22.4-1 (88 and 95%, respectively; Table S2).

MobA from HI2019 was most similar to the characterized MobA proteins from *E. coli* (39% identity, 60% similarity) and *R. sphaeroides* (38% identity, 54% similarity; Figure 1B, Table S3), with slightly lower identity values being obtained for MobA from *R. capsulatus* (31% identity, 55% similarity) and *P. aeruginosa* (29% identity, 46% similarity; Figure 1B).

Several conserved sequence motifs (residues 12–25, 78–82, 100–103, and 176–182) involved in the binding of GTP (N-terminal) and Mo-MPT (C-terminal; Lake et al., 2000) mediate the function of MobA, and those sequence motifs are also present in *H. influenzae* MobA proteins (Figure 1B and Figure S2). Interestingly, we observed that in 6 out of 25 *H. influenzae* MobA sequences obtained from GenBank a major part (residues 10–17) of the first conserved sequence motif appeared to be missing (Figure S2). Our analyses of the *mobA* gene regions of strains NTHI PittGG and NTHI PittEE revealed that in both cases the *mobA* start codon had been wrongly predicted, and that the *mobA* gene was actually longer than annotated in the genome and the additional sequence part included the “missing” first sequence motif. We assume that this is the case for the other four strains as well. Taken together, the above data suggest that MobA from *H. influenzae* is functional and supports MGD synthesis.

### The MobA protein is essential for molybdenum enzymes activities in NTHI

To investigate the role of MobA in NTHI, a knockout mutation of *mobA* was created in HI2019, a strain isolated from a COPD patient (Campagnari et al., 1987). The mutant strain was complemented by insertion of a functional copy of *mobA* into ORF*HI601.1* (Johnston et al., 2007). Following PCR verification of the strain genotypes, enzyme assays were used to investigate the effect of the *mobA* mutation on the activity of HI molybdenum enzymes. DMSO reductase (DMSOR), nitrate reductase (NR), and MetSO reductase (MetSOR) activities were tested in cell extracts from anaerobically grown cultures, while formate dehydrogenase (FDH) was assayed in cell extracts from cultures grown under microaerophilic conditions since under these conditions the expression of those enzymes is maximal (Othman et al., 2014).

**Figure 2:**
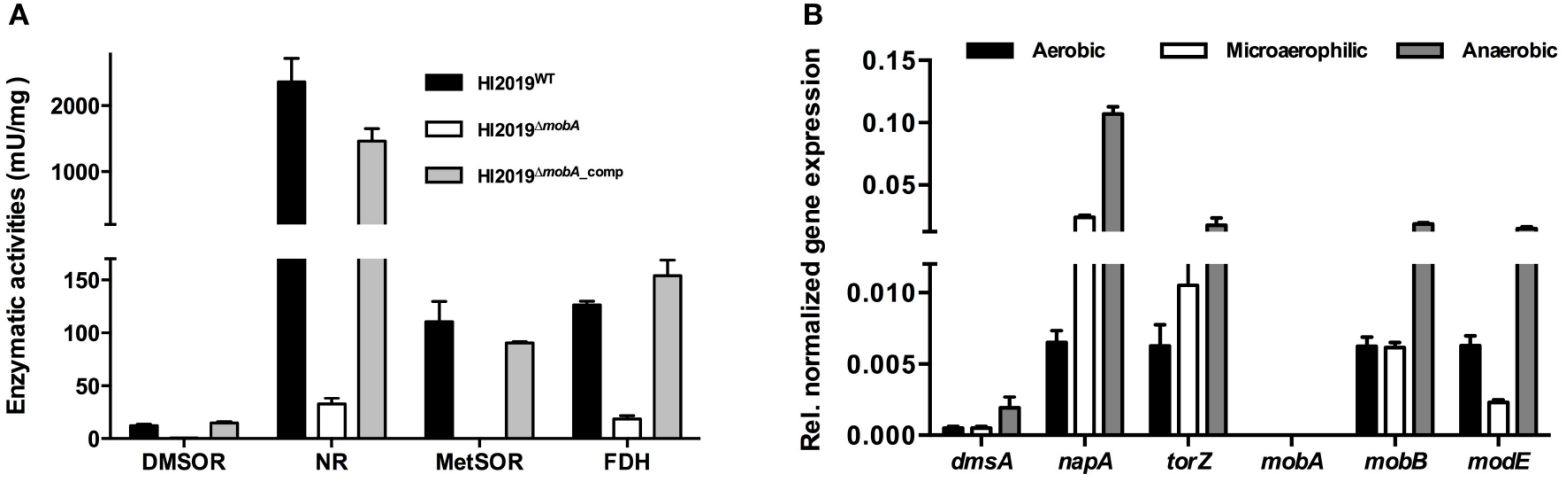
**Assessing the effect of a deletion of ***mobA*** gene on molybdenum enzymes activities and gene expression in NTHI**. **(A)** Enzyme activity of different molybdenum enzymes in HI2019^WT^, HI2019^Δ*mobA*^, and HI2019^Δ*mobA*_comp^ strains. Reductase activities were analyzed in crude extracts after anaerobic growth of HI2019^WT^, HI2019^Δ*mobA*^, and HI2019^Δ*mobA*_comp^ strains in sRPMI. DMSO reductase (DMSOR), nitrate reductase (NR), and MetSO reductase (MetSOR) activities were expressed as μmol of DMSO, nitrate, and MetSO reduced per min (U) per mg of proteins, respectively. Formate dehydrogenase (FDH) activity was assayed in crude extracts after microaerophilic growth and was expressed as μ mol of formate oxidized per min (U) per mg of proteins.**(B)** Expression of genes encoding or involved in molybdenum enzymes activities in HI2019^Δ*mobA*^ grown under aerobic, microaerophilic, and anaerobic conditions. *dmsA*, DMSOR; *torZ*, putative TMAO reductase; *napA*, NR; *modE*, activator of the transcription of genes involved in cellular molybdenum metabolism.

In HI2019^WT^ cell extracts, DMSOR and MetSOR activities were 12.2 ± 1.5 mU/mg and 110.5 ± 19.2 mU/mg, respectively, while NR activity (2361 ± 353 mU/mg) clearly exceeded the DMSOR (200 ×) and MetSOR (20 ×) activities (Figure 2A). FDH activity in the WT strain was 126.5 ± 3.4 mU/mg. The absence of a functional copy of *mobA* led to a complete loss of MetSOR activity and a significant reduction of the FDH, DMSOR, and NR activities by 85, 95, and 99%, respectively (Figure 2A). Those activities were, however, restored to WT levels by the complementation of the *mobA* mutation (DMSOR: 14.9±1.1 mU/mg, METSOR: 90.0 ± 1.2 mU/mg, NR: 1463.9±187 mU/mg, FDH: 153.9±14.9 mU/mg; Figure 2A). Slight variations in the exact expression levels of these enzymes may be due to a combination of factors, including the complex maturation processes and varying affinities for MobA (Magalon et al., 2011).

The observed changes in enzyme activities were not due to a loss of expression of the relevant genes as shown by qRT-PCR (Figure 2B): *dmsA* (DMSOR), *napA* (NR), *torZ* (putative TMAO reductase) as well as *modE* (activator of the transcription of genes involved in cellular molybdenum metabolism) all showed expression levels that were similar to the wild type and also in accordance with our previous results (Othman et al., 2014). Expression of the *mobB* gene was not affected by the loss of *mobA* (Figure 2B) consistent with the fact that the two genes are not forming a transcriptional unit in *H. influenzae*, while no expression of *mobA* was observed as expected in a HI2019^Δ*mobA*^ strain. These results confirm that in HI2019, the mutation of the *mobA* gene exerts a pleiotropic effect on all molybdenum enzyme activities as has also been reported for other bacteria (Johnson et al., 1991; Leimkuhler and Klipp, 1999; Buchanan et al., 2001), and that we successfully managed to complement the knockout mutation.

### MobA is important for anaerobic growth of HI2019

**Figure 3:**
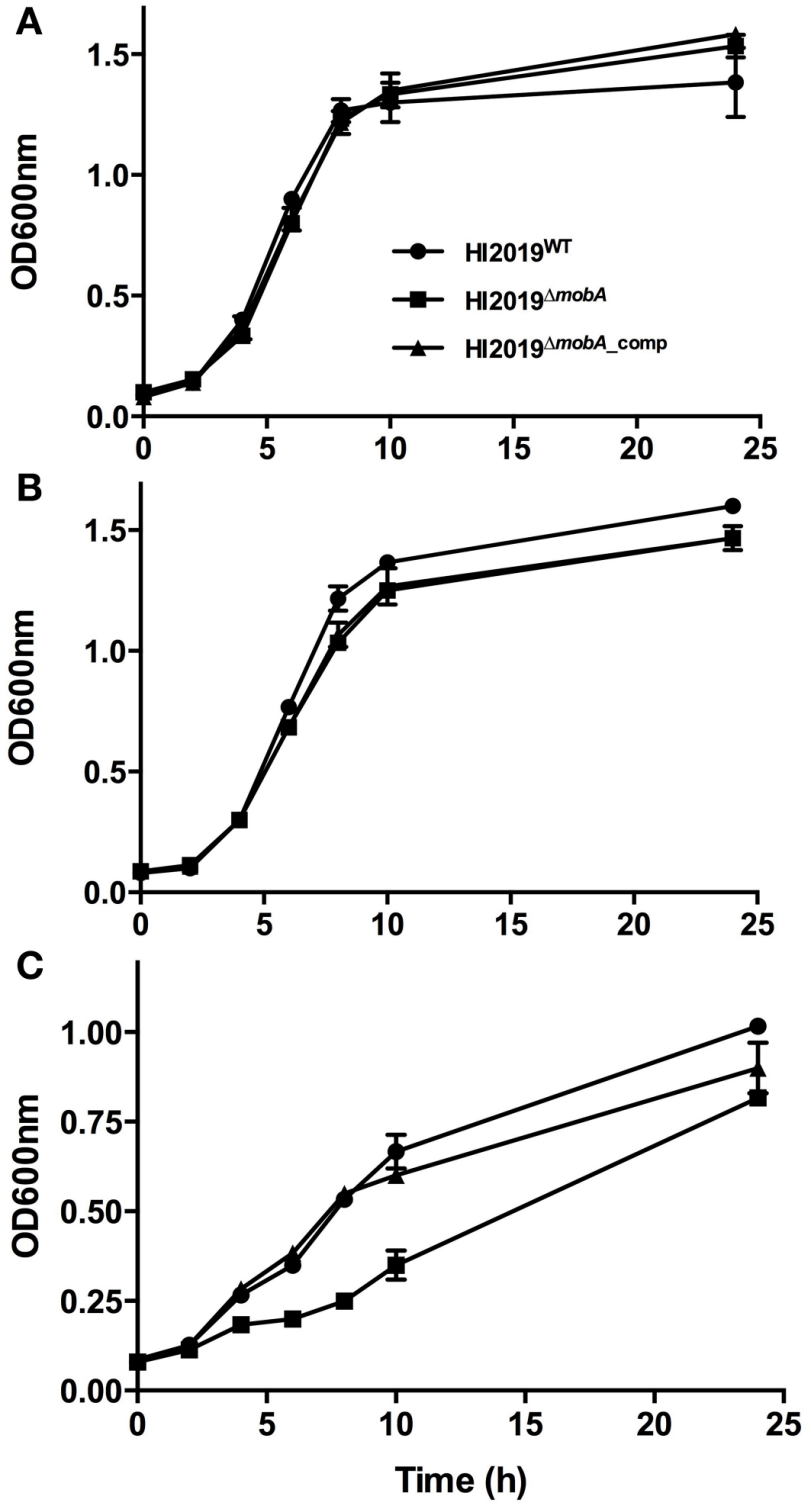
**Growth of HI2019^WT^, HI2019^Δ***mobA***^, and HI2019^Δ***mobA***_comp^ strains in the presence of different oxygen tensions**. Aerobic **(A)**, microaerophilic **(B),** and anaerobic **(C)** growth of HI2019^WT^ (full circle), HI2019^Δ*mobA*^ (full square) and HI2019^Δ*mobA*_comp^ (full triangle) strains has been monitored using optical density of cultures at 600 nm. sRPMI was used as a culture medium.

HI2019^WT^, HI2019^Δ*mobA*^, and HI2019^Δ*mobA*_comp^ strains were tested for their ability to grow under aerobic, microaerophilic, and anaerobic conditions on sRPMI medium. For aerobic and microaerophilic growth, all three strains showed identical growth behavior (Figure 3). However, under anaerobic conditions, the HI2019^Δ*mobA*^ strain had a growth rate of 0.10 h^−1^ (doubling time = 6.89 h), about 40% lower than the wild-type (growth rate = 0.17 h^−1^, doubling time = 3.91 h; Figure 3C). Moreover, the final OD_600_ value was only 0.8, a 20% reduction relative to the WT strain. Again, complementation restored the wild-type phenotype (Figure 3C). This finding suggests that MobA is important for anaerobic growth of NTHI but not essential which is similar to observations made for a *P. aeruginosa*^Δ*mobA*^ strain, however, in that case only minimal growth under anaerobic conditions was observed (Noriega et al., 2005).

### Is a functional copy of MobA required for biofilm formation and interaction of NTHI with host cells?

A key factor for NTHI colonization of the human body is biofilm formation (Starner et al., 2006; Swords, 2012). As those biofilms become easily depleted in oxygen (Werner et al., 2004), it seemed likely that the HI2019^Δ*mobA*^ strain might be affected in biofilm formation, as it showed a growth defect under anaerobic conditions. However, using a standard microtitre plate biofilm assay, HI2019^WT^ and HI2019^Δ*mobA*^ formed similar amounts of biofilms under anaerobic conditions (Figure S3).

**Figure 4:**
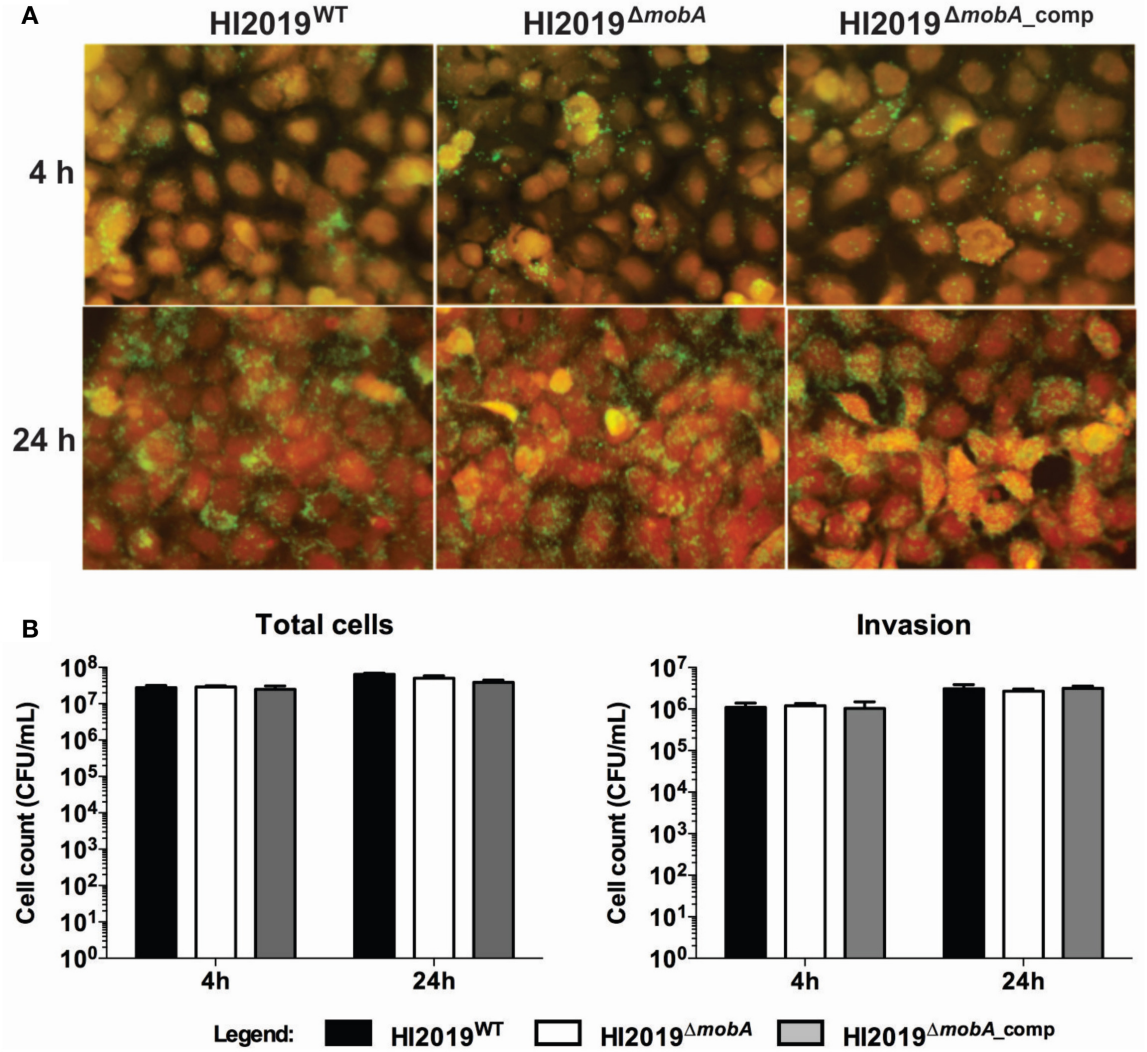
**Interaction of HI2019 strains with human epithelial cells 16HBE14**. **(A)** Immunofluorescence microscopy of 16HBE14 cells infected with HI2019 strains. The epithelial cells were infected with HI2019^WT^, HI2019^Δ*mobA*^, and HI2019^Δ*mobA*_comp^ for 4 h or 24 h with an MOI of 100. Bacteria were stained with anti-mouse IgG (whole molecule)-FITC antibody (green) interacting with the primary antibody 6E4 raised to bacterium surface components. The host cells were stained with CellTracker™ Orange CMTMR fluorescent dye (red). **(B)** Quantification of total cell-associated and internalized bacteria. Epithelial cells (16HBE14) were infected with HI2019^WT^, HI2019^Δ*mobA*^, and HI2019^Δ*mobA*_comp^ strains for 4 or 24 h. The number of bacteria associated and internalized over time was estimated by plating serial dilutions and counting CFU. Data for both internalized and total cell adherent cells did not show any statistically significant differences.

Another key process in establishing NTHI infection in the human body is the colonization of epithelial cells, and an important step in this process is the adherence of bacteria to the host cells (Clementi and Murphy, 2011) which is also the key to NTHI internalization (Clementi and Murphy, 2011). Interestingly, one of the four Mo-enzymes present in *H. influenzae* has been implicated in host cell interactions as it was found that the promoter of the *dmsA* gene showed increased expression under these conditions (van Ulsen et al., 2002). Human bronchial epithelial cells (16HBE14) were co-cultured with the HI2019^WT^, HI2019^Δ*mobA*^, or HI2019^Δ*mobA*_comp^ strains for 4 and 24 h with an initial MOI of 100, stained and then analyzed by immunofluorescence microscopy. Association of HI2019^WT^ with the host cells could be observed within 4 h of infection (Figure 4A), and after 24 h, the number of bacteria found on the cell surface had clearly increased. Neither the mutation in the *mobA* gene nor its complementation appeared to affect the ability of HI2019 to interact with the epithelial cells (Figure 4A) in this assay.

These findings were corroborated by adherence and invasion assays, where, again, all three strains were able to associate with, invade, and survive within 16HBE14 cells after 4 and 24 h incubation (Figure 4B). For HI2019^WT^, the number of total associated bacteria increased about 2.5 times between the 4 and the 24 h time points to a value of 6.5^*^10^7^ ± 5.1^*^10^6^ CFU/mL. Internalized bacteria represented ~4.7% of the total cell-associated bacteria (Figure 4B) at both time points. The HI2019^Δ*mobA*^ strain showed the same behavior as the WT strain with total cell numbers equal to 5.1 ^*^10^7^ ± 8.2^*^10^6^ CFU/mL and 2.7 ^*^10^6^ ± 3.3^*^10^5^ CFU/mL of internalized cells (5.3% of total cells) after 24 h incubation. As could be expected, results for the HI2019^Δ*mobA*_comp^ strain were similar to those obtained for the two other strains, HI2019^WT^ and HI2019^Δ*mobA*^.

**Figure 5:**
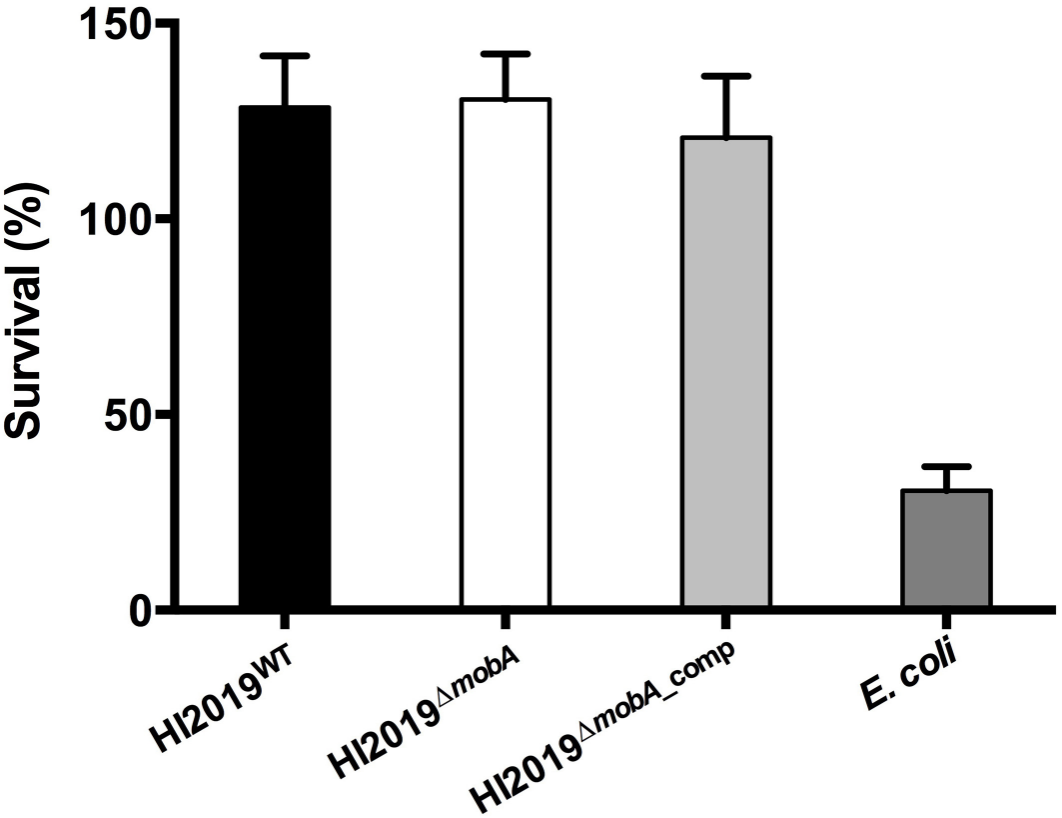
**Neutrophil-dependent killing of HI2019 strains and ***E. coli*** Dh5α**. Neutrophils (2^*^10^∧6^ cells/mL) were incubated with 1:10 HI2019^WT^, HI2019^Δ*mobA*^, and HI2019^Δ*mobA*_comp^ strains and after 2 h of incubation, viable bacteria were measured by CFUs counting as described in Materials and Methods. Values shown are % viable bacteria at 2 h compared to the initial inoculum. *E. coli* Dh5α cells were used as a control.

Another type of host cell that pathogenic bacteria encounter regularly during infection are neutrophils. Using isolated neutrophils, we compared the efficiency of killing of HI2019^WT^, HI2019^Δ*mobA*^, and HI2019^Δ*mobA*_comp^ strains as well as *E. coli* by neutrophils. Approximately 70% of the initial inoculum of *E. coli* DH5α were killed following incubation for 2 h at a 1:10 neutrophils: bacteria ratio (Figure 5). In contrast, under identical experimental conditions, neutrophils failed to kill any of the three HI strains. In fact, the HI strains even appeared to grow in the presence of the neutrophils as the cell numbers detected following 2 h of incubation exceeded those present in the inoculum by ~ 30%.

In conclusion, we can say that while a mutation of the *mobA* gene in NTHI clearly affected the activities of the molybdenum enzymes found in NTHI and also caused a mild growth defect under anaerobic conditions, the interactions between the HI2019^Δ*mobA*^ strain and epithelial cells or neutrophils did not show any changes relative to the WT strain.

### NTHI carrying a mutation in *MobA* showed a colonization defect in a mouse model of infection

**Figure 6:**
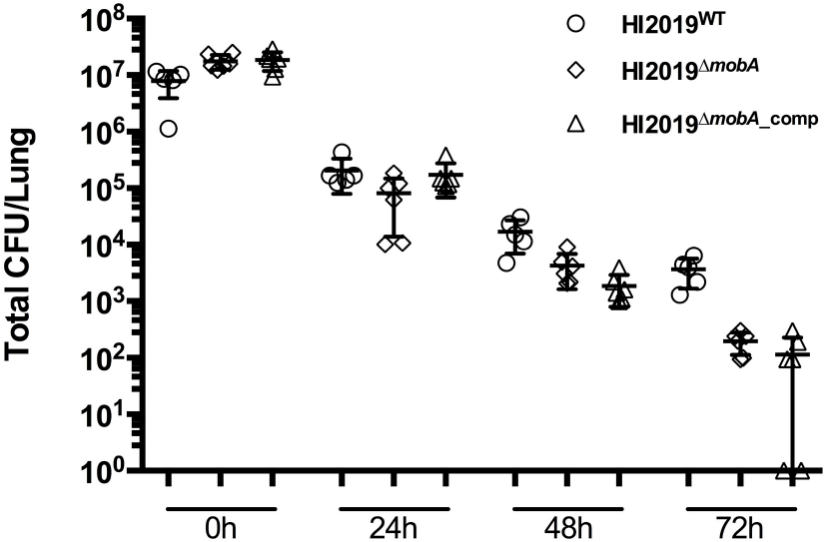
**Survival of HI2019^WT^, HI2019^Δ***mobA***^, and HI2019^Δ***mobA***_comp^ in a murine clearance model**. **(A)** Survival of HI2019 strains. BALB/c mice were infected with 10^7^ CFU of HI2019^WT^, HI2019^Δ*mobA*^, or HI2019^Δ*mobA*_comp^. Bacterial recovery was monitored at 24 h intervals for 72 h in lung homogenates.

Growth conditions imposed during *in vitro* experiments can only partially recreate the much more complex host environment, where molybdenum enzymes may play an essential role in bacterial survival. We therefore investigated whether the mobA mutation affected the fitness of the HI2019^Δ*mobA*^ strain in a mouse clearance model (Morey et al., 2013). Mice were infected with 10^7^ CFU of HI2019^WT^, HI2019^Δ*mobA*^, or HI2019^Δ*mobA*_*comp*^ and bacterial recovery was monitored at 24 h intervals for 72 h (Essilfie et al., 2011). No significant difference in recovered bacterial number was observed at 0 and 24 h post-infection (Figure 6), while after 48 and 72 h a small, but still not statistically significant reduction in recovered bacteria for the three strains was observed (Figure 6).

## Discussion

The observation that bacterial molybdenum enzymes of the DMSO reductase family require the activity of an enzyme not found in eukaryotes has led to the suggestion that MobA might be a suitable target for antibacterials (Anishetty et al., 2005; Williams et al., 2014). Results from studies investigating individual molybdenum enzymes thus far suggest that this might have some merit. For example, a tetrathionate reductase (*ttrA*) mutant of *Salmonella enterica* sv Typhimurium failed to establish itself in a mouse model of gut infection in a competition assay with the wild-type strain (Winter et al., 2010).

While studying *H. influenzae* carrying a mutation in the *mobA* gene, we observed the expected pleiotropic loss of molybdenum enzyme activities as well as a mild growth defect of the mutant strain under anaerobic conditions. These phenotypes were expected based on previous work in other bacteria, and could be fully reversed following complementation of the mutation.

However, while there was a clear phenotype for the HI2019^Δ*mobA*^ strain in growth experiments and enzyme assays, all of our investigations of the interactions of HI2019^Δ*mobA*^ with host cells failed to show substantial changes, with the exception of a small, but not significant defect in persistence in a murine clearance model of infection. Thus, it has to be concluded that in *H. influenzae* the MobA protein is clearly not a good target for drug development.

It had been speculated that by impairing the function of all Mo-MGD enzymes through a *mobA* mutation, an even more severe phenotype could be produced (Contreras et al., 1997; Aly et al., 2006; Rosas-Magallanes et al., 2007). However, this is clearly not the case, and in fact our observations match those made recently for *M. tuberculosis*, where an Mtb^Δ*mob*A^ strain showed no survival defect in human monocytes or in a murine lung infection model (Williams et al., 2015). Interestingly, the same strain of *M. tuberculosis* showed lower levels of survival in guinea pigs after 60 days of incubation, but not after 30 days of incubation (Williams et al., 2015). These results are very similar to our data; we were unable to detect a defect in *in vitro* interactions with host cells for the HI2019^Δ*mobA*^ mutant strain, but then observed a small change in the strain's ability to survive in a murine model of lung infection. This further supports our conclusion that despite impressive phenotypes observed for mutations in individual Mo enzymes, the cofactor synthesis protein, MobA, is not a universal drug target.

It is possible that for bacteria with appreciable plasticity in their metabolic and respiratory pathways such as NTHI, it is less likely that the bacterial pathogen will encounter conditions where molybdenum enzymes have a critical role for survival. As a result, we cannot rule out the possibility that molybdenum enzymes in NTHI might be more important for infection in systems that are more anaerobic (such a otitis media), as three of the four Mo-enzymes affected by the *mobA* knock out mutation should support anaerobic respiration.

The molecular basis for our observations as well as the related work carried out in *M. tuberculosis* is unknown at present, but a link to more global regulatory phenomena triggered by the accumulation of intermediates of the molybdenum cofactor that are the consequence of a mutation in the *mobA* gene cannot be excluded at this stage. The accumulation of molybdenum cofactor intermediates is known to affect the translation of mRNAs involved in molybdenum metabolism and homeostasis via a riboswitch mechanism in *E. coli* (Regulski et al., 2008), and bioinformatics analyses have shown that *H. influenzae* Moco biosynthesis genes also carry sequences related to this P3 riboswitch (Regulski et al., 2008).

## Author contributions

RD and MO carried out the majority of the experimental work, AE and PH realized and collected the mouse infection data, JH advised on appropriate statistical data analysis, AM and UK were responsible for the study design and experimental work. All authors contributed to the writing of the manuscript.

### Conflict of interest statement

The authors declare that the research was conducted in the absence of any commercial or financial relationships that could be construed as a potential conflict of interest.
