# Supplementary material for: Maturation of molybdoenzymes and its influence on the pathogenesis of non-typeable Haemophilus influenzae
Source: Front Microbiol. 2015 Nov 5;6:1219. doi: 10.3389/fmicb.2015.01219 (PMC4633490; doi:10.3389/fmicb.2015.01219)
Supplement: Supplementary file 1 [file SupplementaryTables.PDF]

Supplementary data

Table S1 Presence of *mobA* and *mobB* genes in stains of *H. influenzae*:

| *H. influenzae* strains | *mobA* | *mobB* |
|---|---|---|
| 2019 | √ | √ |
| Rd KW-20 | √ | √ |
| 10810 | √ | √ |
| 86-028NP | √ | √ |
| R2846 | √ | √ |
| 22.1-21 | √ | √ |
| R2866 | √ | √ |
| RdAW | √ | √ |
| 22.4-21 | √ | √ |
| KR494 | √ | √ |
| NT127 | √ | √ |
| R3021 | √ | √ |
| F3031 | √ | √ |
| F3047 | √ | √ |
| 3655 | √ | √ |
| 7P49H1 | √ | √ |
| PittGG | √ | √ |
| CGSHiCZ412602 | √ | √ |
| PittAA | √ | √ |
| PittHH | √ | √ |
| PittII | √ | √ |
| 6P18H1 | √ | √ |
| PittEE | √ | √ |
| 723 | √ | √ |
| C486 | √ | √ |

## Table S2:

MobA amino acid sequence identities for sequences from various *H. influenzae* strains. Values represent identity in percent.

| | HI2019 | HIRdKW20 | HI10810 | HI86-028NP | HIR2846 | HI22.1-21 | HIR2866 | HIRdAW | HI22.4-21 | HIKR494 | HINT127 | HIR3021 | HIF3031 | HIF3047 | HI3655 | HI7P49H1 | HIPittGG | HICGSHiC2412602 | HIPittAA | HIPittHH | HIPittII | HI6P18H1 | HIPittEE | HI723 | HIC486 |
|---|---|---|---|---|---|---|---|---|---|---|---|---|---|---|---|---|---|---|---|---|---|---|---|---|---|
| HI2019 | | 99 | 99 | 100 | 99 | 98 | 98 | 99 | 90 | 93 | 97 | 98 | 98 | 98 | 99 | 99 | 99 | 99 | 99 | 99 | 98 | 99 | 99 | 100 | 99 |
| HIRdKW20 | | | 99 | 99 | 99 | 99 | 99 | 100 | 90 | 93 | 97 | 98 | 98 | 98 | 99 | 99 | 99 | 99 | 99 | 99 | 99 | 99 | 99 | 99 | 99 |
| HI10810 | | | | 99 | 99 | 98 | 98 | 99 | 90 | 93 | 98 | 98 | 98 | 98 | 99 | 99 | 99 | 99 | 100 | 99 | 98 | 99 | 99 | 99 | 99 |
| HI86-028NP | | | | | 99 | 98 | 98 | 99 | 90 | 93 | 97 | 98 | 98 | 98 | 99 | 99 | 99 | 99 | 99 | 99 | 98 | 99 | 99 | 100 | 99 |
| HIR2846 | | | | | | 98 | 98 | 99 | 90 | 93 | 98 | 98 | 99 | 99 | 99 | 99 | 100 | 100 | 100 | 99 | 98 | 99 | 99 | 99 | 99 |
| HI22.1-21 | | | | | | | 100 | 99 | 90 | 92 | 97 | 97 | 97 | 97 | 98 | 98 | 98 | 98 | 98 | 98 | 100 | 98 | 98 | 98 | 98 |
| HIR2866 | | | | | | | | 99 | 90 | 92 | 97 | 97 | 97 | 97 | 98 | 98 | 98 | 98 | 98 | 98 | 100 | 98 | 98 | 98 | 98 |
| HIRdAW | | | | | | | | | 90 | 93 | 97 | 98 | 98 | 98 | 99 | 99 | 99 | 99 | 99 | 99 | 99 | 99 | 99 | 99 | 99 |
| HI22.4-21 | | | | | | | | | | 93 | 90 | 91 | 90 | 90 | 91 | 91 | 90 | 90 | 90 | 89 | 88 | 90 | 91 | 90 | 91 |
| HIKR494 | | | | | | | | | | | 94 | 95 | 93 | 93 | 93 | 93 | 93 | 93 | 92 | 92 | 91 | 92 | 93 | 93 | 93 |
| HINT127 | | | | | | | | | | | | 99 | 97 | 97 | 97 | 97 | 98 | 98 | 98 | 98 | 97 | 98 | 97 | 97 | 97 |
| HIR3021 | | | | | | | | | | | | | 97 | 97 | 98 | 98 | 98 | 98 | 98 | 98 | 97 | 98 | 98 | 98 | 98 |
| HIF3031 | | | | | | | | | | | | | | 100 | 98 | 98 | 99 | 99 | 99 | 98 | 97 | 98 | 98 | 98 | 98 |
| HIF3047 | | | | | | | | | | | | | | | 98 | 98 | 99 | 99 | 99 | 98 | 97 | 98 | 98 | 98 | 98 |
| HI3655 | | | | | | | | | | | | | | | | 100 | 99 | 99 | 99 | 99 | 98 | 100 | 100 | 99 | 100 |
| HI7P49H1 | | | | | | | | | | | | | | | | | 99 | 99 | 99 | 99 | 98 | 100 | 100 | 99 | 100 |
| HIPittGG | | | | | | | | | | | | | | | | | | 100 | 100 | 99 | 98 | 99 | 99 | 99 | 99 |
| HICGSHiC2412602 | | | | | | | | | | | | | | | | | | | 100 | 99 | 98 | 99 | 99 | 99 | 99 |
| HIPittAA | | | | | | | | | | | | | | | | | | | | 99 | 98 | 99 | 99 | 99 | 99 |
| HIPittHH | | | | | | | | | | | | | | | | | | | | | 98 | 99 | 99 | 99 | 99 |
| HIPittII | | | | | | | | | | | | | | | | | | | | | | 98 | 98 | 98 | 98 |
| HI6P18H1 | | | | | | | | | | | | | | | | | | | | | | | 100 | 99 | 100 |
| HIPittEE | | | | | | | | | | | | | | | | | | | | | | | | 99 | 100 |
| HI723 | | | | | | | | | | | | | | | | | | | | | | | | | 99 |
| HIC486 | | | | | | | | | | | | | | | | | | | | | | | | | |

# Table S3:

MobB amino acid sequence identities fro sequences from various *H. influenzae* strains. Values represent identity in percent.

| | HI2019 | HIRdKW20 | HI10810 | HI86-028NP | HIR2846 | HI22.1-21 | HIR2866 | HIRdAW | HI22.4-21 | HIKR494 | HINT127 | HIR3021 | HIF3031 | HIF3047 | HI3655 | HI7P49H1 | HIPittGG | HICGSHiCZ412602 | HIPittAA | HIPittHH | HIPittII | HI6P18H1 | HIPittEE | HIC486 | HI723 |
|---|---|---|---|---|---|---|---|---|---|---|---|---|---|---|---|---|---|---|---|---|---|---|---|---|---|
| HI2019 | | 98 | 98 | 99 | 96 | 98 | 98 | 98 | 97 | 96 | 98 | 96 | 96 | 96 | 96 | 96 | 98 | 96 | 96 | 96 | 98 | 96 | 96 | 96 | 99 |
| HIRdKW20 | | | 99 | 99 | 98 | 99 | 99 | 100 | 99 | 98 | 99 | 98 | 98 | 98 | 98 | 98 | 99 | 98 | 98 | 98 | 99 | 98 | 98 | 98 | 99 |
| HI10810 | | | | 98 | 99 | 98 | 98 | 99 | 98 | 97 | 99 | 97 | 97 | 98 | 99 | 99 | 98 | 99 | 98 | 98 | 98 | 99 | 99 | 99 | 98 |
| HI86-028NP | | | | | 97 | 99 | 99 | 99 | 98 | 96 | 98 | 96 | 96 | 97 | 97 | 97 | 99 | 97 | 96 | 97 | 99 | 97 | 97 | 97 | 100 |
| HIR2846 | | | | | | 98 | 98 | 98 | 98 | 97 | 99 | 97 | 97 | 98 | 100 | 100 | 98 | 100 | 99 | 97 | 98 | 100 | 100 | 100 | 97 |
| HI22.1-21 | | | | | | | 100 | 99 | 98 | 96 | 98 | 96 | 96 | 97 | 98 | 98 | 100 | 98 | 98 | 97 | 100 | 98 | 98 | 98 | 99 |
| HIR2866 | | | | | | | | 99 | 98 | 96 | 98 | 96 | 96 | 97 | 98 | 98 | 100 | 98 | 98 | 97 | 100 | 98 | 98 | 98 | 99 |
| HIRdAW | | | | | | | | | 99 | 98 | 99 | 98 | 98 | 98 | 98 | 98 | 99 | 98 | 98 | 98 | 99 | 98 | 98 | 98 | 99 |
| HI22.4-21 | | | | | | | | | | 98 | 99 | 98 | 98 | 98 | 98 | 98 | 98 | 98 | 98 | 98 | 98 | 98 | 98 | 98 | 98 |
| HIKR494 | | | | | | | | | | | 98 | 99 | 96 | 97 | 97 | 97 | 96 | 97 | 96 | 98 | 96 | 97 | 97 | 97 | 96 |
| HINT127 | | | | | | | | | | | | 98 | 98 | 99 | 99 | 99 | 98 | 99 | 98 | 98 | 98 | 99 | 99 | 99 | 98 |
| HIR3021 | | | | | | | | | | | | | 96 | 97 | 97 | 97 | 96 | 97 | 96 | 99 | 96 | 97 | 97 | 97 | 96 |
| HIF3031 | | | | | | | | | | | | | | 98 | 97 | 97 | 96 | 97 | 96 | 96 | 96 | 97 | 97 | 97 | 96 |
| HIF3047 | | | | | | | | | | | | | | | 98 | 98 | 97 | 98 | 97 | 97 | 97 | 98 | 98 | 98 | 97 |
| HI3655 | | | | | | | | | | | | | | | | 100 | 98 | 100 | 99 | 97 | 98 | 100 | 100 | 100 | 97 |
| HI7P49H1 | | | | | | | | | | | | | | | | | 98 | 100 | 99 | 97 | 98 | 100 | 100 | 100 | 97 |
| HIPittGG | | | | | | | | | | | | | | | | | | 98 | 98 | 97 | 100 | 98 | 98 | 98 | 99 |
| HICGSHiCZ412602 | | | | | | | | | | | | | | | | | | | 99 | 97 | 98 | 100 | 100 | 100 | 97 |
| HIPittAA | | | | | | | | | | | | | | | | | | | | 96 | 98 | 99 | 99 | 99 | 96 |
| HIPittHH | | | | | | | | | | | | | | | | | | | | | 97 | 97 | 97 | 97 | 97 |
| HIPittII | | | | | | | | | | | | | | | | | | | | | | 98 | 98 | 98 | 99 |
| HI6P18H1 | | | | | | | | | | | | | | | | | | | | | | | 100 | 100 | 97 |
| HIPittEE | | | | | | | | | | | | | | | | | | | | | | | | 100 | 97 |
| HIC486 | | | | | | | | | | | | | | | | | | | | | | | | | 97 |
| HI723 | | | | | | | | | | | | | | | | | | | | | | | | | |
