# Supplementary material for: Maturation of molybdoenzymes and its influence on the pathogenesis of non-typeable Haemophilus influenzae
Source: Front Microbiol. 2015 Nov 5;6:1219. doi: 10.3389/fmicb.2015.01219 (PMC4633490; doi:10.3389/fmicb.2015.01219)
Supplement: Supplementary file 2 [file SupplementaryFigures.PDF]

Figure S1:

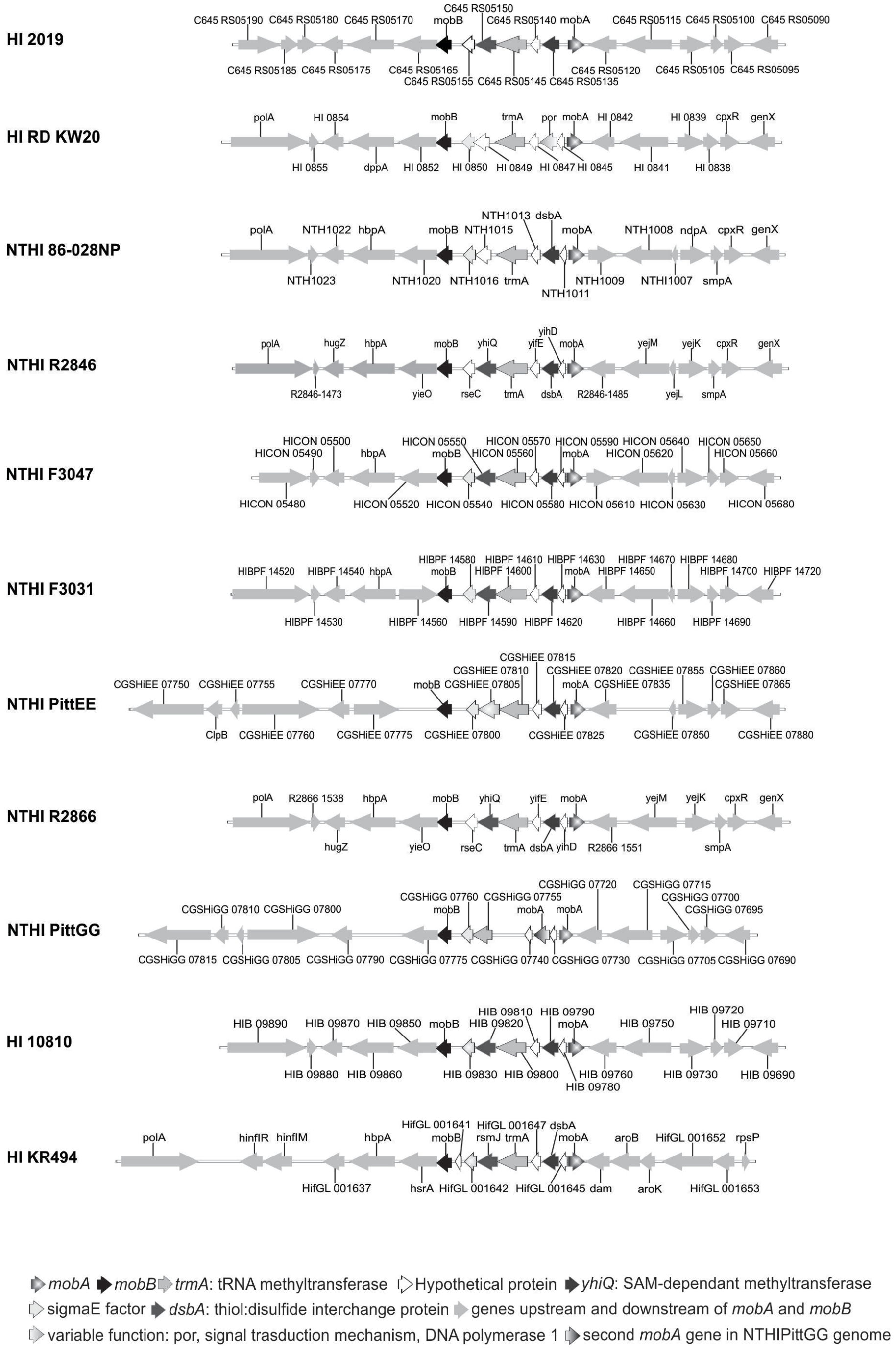

Genomic comparison of *mob* locus in different *H. influenzae* strains with complete genomes: HI2019, HI RdKW20, 7 other NTHI strains (NTHI 86-028NP, NTHI R2846, NTHI F3047, NTHI F3031, NTHI PittEE, NTHI R2866 and NTHI PittGG) and 2 *H. influenzae* serotype b and f strains (HI 10810 and HI KR494).

Figure S2:

```
                    1                                                                                                 100
         HI2019    (1) MTITISAVILAGGKARRMGGQDKGLQILGKQSLIEHIINRLQPQIHQISINTNRNQTEYAKFGFPVFSDELPDFQGPLSGMLTALEKTKSDFILFTPQDT
        HIRdKW20   (1) MTITISAVILAGGKARRMGGQDKGLQILGKQSLIEHVINRLQPQIHQISINANRNQTEYAKFGFPVFSDELPDFQGPLSGMLTALEKTKSDFILFTPQDT
         HI10810   (1) MTITISSVILAGGKARRMGGQDKGLQILGKQSLIEHVINRLQPQIHQISINANRNQTEYAKFGFPVFSDELPDFQGPLSGMLTALEKTKSDFILFTPQDT
      HI86-028NP   (1) MTITISAVILAGGKARRMGGQDKGLQILGKQSLIEHVINRLQPQIHQISINTNRNQTEYAKFGFPVFSDELPDFQGPLSGMLTALEKTKSDFILFTPQDT
         HIR2846   (1) MTITISAVILAGGKARRMGGQDKGLQILGKQSLIEHVINRLQPQIHQISINANRNQTEYAKFGFPVFSDELPDFQGPLSGMLTALEKTKSDFILFTPQDT
       HI22.1-21   (1) MTITISAVILAGGKARRMGGQDKGLQILGKQSLIEHIINRLQPQIHQISINANRNQTEYAKFGFPVFSDELPDFQGPLSGMLTALEKTKSDFILFTPQDT
         HIR2866   (1) MTITISAVILAGGKARRMGGQDKGLQILGKQSLIEHIINRLQPQIHQISINANRNQTEYAKFGFPVFSDELPDFQGPLSGMLTALEKTKSDFILFTPQDT
          HIRdAW   (1) MTITISAVILAGGKARRMGGQDKGLQILGKQSLIEHIINRLQPQIHQISINANRNQTEYAKFGFPVFSDELPDFQGPLSGMLTALEKTKSDFILFTPQDT
       HI22.4-21   (1) MTITISAVILAGGKARRMGGQDKGLQILGKQPLIQYVINRLQPQLHDISINANRSQTEYAKFGFPIFSDELPDFQGPLSGMLTALEKTKSDFILFTPQDT
         HIKR494   (1) MTITISAVILAGGKARRMGEQDKGLQILGKQSLIQHVINRLQPQVHDISINANRSQTEYAKFGFPVFSDELPDFQGPLSGMLTALEKTKSDFILFTPQDT
         HINT127   (1) MTITISTVILAGGKARRMGGQDKGLQILGKQSLIEHVINRLQPQIHQISINANRNQTEYAKFGFPVFSDELPDFQGPLSGMLTALEKTKSDFILFTPQDT
         HIR3021   (1) MTITISAVILAGGKARRMGGQDKGLQILGKQSLIEHVINRLQPQIHQISINANRNQTEYAKFGFPVFSDELPDFQGPLSGMLTALEKTKSDFILFTPQDT
         HIF3031   (1) MTITISAVILAGGKARRMGGQDKGLQILGKQSLIEHVIHRLQPQIHQISINANRNQTEYAKFGFPVFSDELPDFQGPLSGMLTALEKTKSDFILFTPQDT
         HIF3047   (1) MTITISAVILAGGKARRMGGQDKGLQILGKQSLIEHVIHRLQPQIHQISINANRNQTEYAKFGFPVFSDELPDFQGPLSGMLTALEKTKSDFILFTPQDT
          HI3655   (1) MTITISAVILAGGKARRMGGQDKGLQILGKQSLIEHVINRLQPQIHQISINANRNQTEYAKFGFPVFSDELPDFQGPLSGMLTALEKTKSDFILFTPQDT
        HI7P49H1   (1) MTITISAVILAGGKARRMGGQDKGLQILGKQSLIEHVINRLQPQIHQISINANRNQTEYAKFGFPVFSDELPDFQGPLSGMLTALEKTKSDFILFTPQDT
        HIPittGG   (1) MTITISAVILAGGKARRMGGQDKGLQILGKQSLIEHVINRLQPQIHQISINANRNQTEYAKFGFPVFSDELPDFQGPLSGMLTALEKTKSDFILFTPQDT
 HICGSHiC2412602   (1) MTITISAVILAGGKARRMGGQDKGLQILGKQSLIEHVINRLQPQIHQISINANRNQTEYAKFGFPVFSDELPDFQGPLSGMLTALEKTKSDFILFTPQDT
        HIPittAA   (1) ----------------MGGQDKGLQILGKQSLIEHVINRLQPQIHQISINANRNQTEYAKFGFPVFSDELPDFQGPLSGMLTALEKTKSDFILFTPQDT
        HIPittHH   (1) ----------------MGGQDKGLQILGKQSLIEHVINRLQPQIHQISINANRNQTEYAKFGFPVFSDELPDFQGPLSGMLTALEKTKSDFILFTPQDT
        HIPittII   (1) ----------------MGGQDKGLQILGKQSLIEHIINRLQPQIHQISINANRNQTEYAKFGFPVFSDELPDFQGPLSGMLTALEKTKSDFILFTPQDT
        HI6P18H1   (1) ----------------MGGQDKGLQILGKQSLIEHVINRLQPQIHQISINANRNQTEYAKFGFPVFSDELPDFQGPLSGMLTALEKTKSDFILFTPQDT
        HIPittEE   (1) MTITISAVILAGGKARRMGGQDKGLQILGKQSLIEHVINRLQPQIHQISINANRNQTEYAKFGFPVFSDELPDFQGPLSGMLTALEKTKSDFILFTPQDT
           HI723   (1) MTITISAVILAGGKARRMGGQDKGLQILGKQSLIEHVINRLQPQIHQISINTNRNQTEYAKFGFPVFSDELPDFQGPLSGMLTALEKTKSDFILFTPQDT
          HIC486   (1) MTITISAVILAGGKARRMGGQDKGLQILGKQSLIEHVINRLQPQIHQISINANRNQTEYAKFGFPVFSDELPDFQGPLSGMLTALEKTKSDFILFTPQDT
       Consensus   (1) MTITISAVILAGGKARRMGGQDKGLQILGKQSLIEHVINRLQPQIHQISINANRNQTEYAKFGFPVFSDELPDFQGPLSGMLTALEKTKSDFILFTPQDT

                       101                                                                                    192
         HI 2019 (101) PFFPMNLLDKLKSAVKNDRTLIAYACDEEREHPVFCLMSVQLKEKLRHYLASGERRLLQFMKENGGISVKFTQEEGNFENFNTLDDLKKTVI
        HIRdKW20 (101) PFFPMNLLDKLKSAVKNDRTLIAYACDEEREHPVFCLMSVQLKEKLRHYLASGERRLLQFMKENGGISVKFTQEEGNFENFNTLDDLKKTVI
         HI10810 (101) PFFPMNLLDKLKSAVKNDRTLIAYACDEEREHPVFCLMSVQLKEKLRHYLASGERRLLQFMKENGGISVKFTQEEGNFENFNTLDDLKKTVI
      HI86-028NP (101) PFFPMNLLDKLKSAVKNDRTLIAYACDEEREHPVFCLMSVQLKEKLRHYLASGERRLLQFMKENGGISVKFTQEEGNFENFNTLDDLKKTVI
         HIR2846 (101) PFFPMNLLDKLKSAVKNDRTLIAYACDEEREHPVFCLMSVQLKEKLRHYLASGERRLLQFMKENGGISVKFTQEEGNFENFNTLDDLKKTVI
       HI22.1-21 (101) PFFPINLLDKLKSAVKNDRTLIVYACDEEREHPVFCLMSVQLKEKLRHYLASGERRLLQFMKENGGISVKFTQEEGNFENFNTLDDLKKTVI
         HIR2866 (101) PFFPINLLDKLKSAVKNDRTLIVYACDEEREHPVFCLMSVQLKEKLRHYLASGERRLLQFMKENGGISVKFTQEEGNFENFNTLDDLKKTVI
          HIRdAW (101) PFFPMNLLDKLKSAVKNDRTLIAYACDEEREHPVFCLMSVQLKEKLRHYLASGERRLLQFMKENGGISVKFTQEEGNFENFNTLDDLKKTVI
       HI22.4-21 (101) PFFPTNLLDKLQSAVKNHRTFIAYACDEEREHPVFCLMSVQLKEQLRHYLASGERRLLQFMKENDGISVKFTKKEGNFENFNTLDDLKGSH-
         HIKR494 (101) PFFPTNLLDKLKSAVENHRTFIAYACDEEREHPVFCLMSVQLKEQLRHYLASGERRLLQFMKENDGISVNFAQEEGNFENFNTLDDLKKTVI
         HINT127 (101) PFFPTNLLDKLKSAVENHRTLIAYACDEEREHPVFCLMSVQLKEKLRHYLASGERRLLQFMKENGGISVKFTQEEGNFENFNTLDDLKKTVI
         HIR3021 (101) PFFPTNLLDKLKSAVENHRTLIAYACDEEREHPVFCLMSVQLKEKLRHYLASGERRLLQFMKENGGISVKFTQEEGNFENFNTLDDLKKTVI
         HIF3031 (101) PFFPMNLLDKLKSAVKNDRTLIAYACDEEREHPVFCLMSVQLKEKLRHYLASGERRLLQFMKENGGISVKFAQEEGNFENFNTLDDLKKTVI
         HIF3047 (101) PFFPMNLLDKLKSAVKNDRTLIAYACDEEREHPVFCLMSVQLKEKLRHYLASGERRLLQFMKENGGISVKFAQEEGNFENFNTLDDLKKTVI
          HI3655 (101) PFFPMNLLDKLKSAVKNDRTLIAYACDEEREHPVFCLMSVQLKEKLRHYLASGERRLLQFMKENGGISVKFTKEEGNFENFNTLDDLKKTVI
        HI7P49H1 (101) PFFPMNLLDKLKSAVKNDRTLIAYACDEEREHPVFCLMSVQLKEKLRHYLASGERRLLQFMKENGGISVKFTKEEGNFENFNTLDDLKKTVI
        HIPittGG (101) PFFPMNLLDKLKSAVKNDRTLIAYACDEEREHPVFCLMSVQLKEKLRHYLASGERRLLQFMKENGGISVKFTQE-GNFENFNTLDDLKKTVI
 HICGSHiC2412602 (101) PFFPMNLLDKLKSAVKNDRTLIAYACDEEREHPVFCLMSVQLKEKLRHYLASGERRLLQFMKENGGISVKFTQEEGNFENFNTLDDLKKTVI
        HIPittAA  (84) PFFPMNLLDKLKSAVKNDRTLIAYACDEEREHPVFCLMSVQLKEKLRHYLASGERRLLQFMKENGGISVKFTQEEGNFENFNTLDDLKKTVI
        HIPittHH  (84) PFFPMNLLDKLKSAVKNDRTLIAYACDEEREHPVFCLMSVQLKEKLRHYLASGERRLLQFMKENGGISVKFTQEESNFENFNTLDDLKKTVI
        HIPittII  (84) PFFPINLLDKLKSAVKNDRTLIVYACDEEREHPVFCLMSVQLKEKLRHYLASGERRLLQFMKENGGISVKFTQEEGNFENFNTLDDLKKTVI
        HI6P18H1  (84) PFFPMNLLDKLKSAVKNDRTLIAYACDEEREHPVFCLMSVQLKEKLRHYLASGERRLLQFMKENGGISVKFTKEEGNFENFNTLDDLKKTVI
        HIPittEE (101) PFFPMNLLDKLKSAVKNDRTLIAYACDEEREHPVFCLMSVQLKEKLRHYLASGERRLLQFMKENGGISVKFTKEEGNFENFNTLDDLKKTVI
           HI723 (101) PFFPMNLLDKLKSAVKNDRTLIAYACDEEREHPVFCLMSVQLKEKLRHYLASGERRLLQFMKENGGISVKFTQEEGNFENFNTLDDLKKTVI
          HIC486 (101) PFFPMNLLDKLKSAVKNDRTLIAYACDEEREHPVFCLMSVQLKEKLRHYLASGERRLLQFMKENGGISVKFTKEEGNFENFNTLDDLKKTVI
       Consensus (101) PFFPMNLLDKLKSAVKNDRTLIAYACDEEREHPVFCLMSVQLKEKLRHYLASGERRLLQFMKENGGISVKFTQEEGNFENFNTLDDLKKTVI
```

Alignment of MobA aminoacid sequences from various *H. influenzae* strains.

Alignments of MobA sequences from 25 *H. influenzae* strains using Vector NTI® software (Life Technologies). Motifs predicted to be required for substrate interactions (Lake et al., 2000) are boxed.

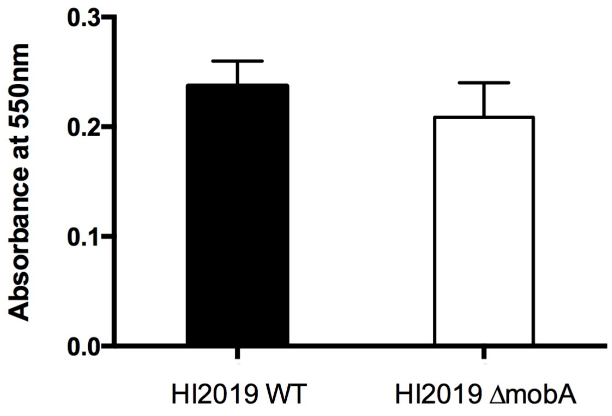

Figure S3:

Biofilm formation of HI2019 WT and HI2019 ΔmobA.

HI2019 WT and HI2019 ΔmobA were grown on polystyrene microtitre plates for 24 h at 37ºC with 5% CO_2. Biofilm formation was determined with a crystal violet assay following a protocol adapted from (Schembri et al., 2001) and reported by absorbance at 550 nm.

Biofilm assays Method

Biofilm assays were performed following a protocol adapted from (Schembri et al., 2001). Briefly, NTHI strains were cultured aerobically on sBHI at 37ºC to an OD_{600nm} of 0.3, diluted to an OD_{600nm} of 0.05 in 96-well microtitre plates (U-bottom, polystyrene, TechnoPlas) and then grown for 24 h at 37 °C in an anaerobic jar. Unattached cells were washed away with sterile water and the bound cells were stained with 0.1% crystal violet for 30 min at room temperature. Excess stain was removed by several washes with sterile water. Crystal violet bound to adherent cells was resuspended by adding 125 µL of 30% acetic acid at room temperature for 15 min. After transferring to a new plate, the biofilm formation was quantified by measuring the absorbance at 550 nm.
